# Sufentanil for Spinal Analgesia during Cesarean Section Delivery: A Systematic Review and Meta-Analysis of Randomized Controlled Trials

**DOI:** 10.1155/2022/4741141

**Published:** 2022-08-31

**Authors:** Hongming Huang, Shiwu Wang, Rujun Lin, Zhongrun He

**Affiliations:** ^1^Department of Anesthesiology, People's Hospital of Wanning, Hainan 571500, China; ^2^Department of Anesthesiology, Chinese Medicine Hospital of Chengmai County, Chengmai 571900, China; ^3^Department of Anesthesiology, Hainan Second People's Hospital, Wuzhishan 572299, China

## Abstract

**Objective:**

To investigate the effect of sufentanil for spinal analgesia during cesarean section.

**Method:**

Eligible papers were systematically retrieved from PubMed, Embase, Ovid, and ScienceDirect. Two researchers independently extracted primary and secondary endpoints to compute relative risk and mean difference by using the random-effects model or the fixed-effects model, as appropriate. Publication bias was quantified and assessed using funnel plot and Egger's test.

**Result:**

A total of 8 publications with 503 pregnant women were included in this study for meta-analysis. Subarachnoid administration of sufentanil did not significantly reduce the onset time of sensory block and motor block. Nonetheless, subarachnoid administration of sufentanil significantly increased the incidence of postoperative skin pruritus (RR = 5.25, 95%CI: 1.90, 14.49, *P* < 0.001).

**Conclusion:**

Subarachnoid administration of sufentanil has no significant difference in the combined effect value of shortening the onset time of sensory block and motor block, prolonging the onset time of local anesthesia and the incidence of some adverse reactions (such as postoperative nausea, vomiting, hypotension, and tremors). However, the incidence of skin pruritus was significantly increased, and the difference was statistically significant. Because of this, the drug still needs to be used with caution in combination with the actual situation in clinical use.

## 1. Introduction

Sufentanil, an opioid analgesic, has been known for its rapid onset of action and potent effect (5–10 times stronger than fentanyl). Its administration route by intravenous, subarachnoid, or subdural injection has been recommended by international guidelines [1]. Sufentanil is considered to be ideal for subdural administration due to its rapid onset of action and lipid solubility, allowing reduced migration and diffusion in the cerebrospinal fluid [2, 3]. Intraspinal administration of sufentanil can significantly reduce the onset time of anesthesia, improve its efficacy, and prolong anesthesia duration [4, 5]. In addition, sufentanil has also been shown to maintain hemodynamic stability similar to that of fentanyl [6]. Moreover, as compared with fentanyl combined with bupivacaine, sufentanil combined with bupivacaine can prolong the total duration of anesthesia, promote rapid sensory blockade, increase oxygen saturation, and reduce the incidence of overall adverse drug reactions. Another study found that sufentanil combined with hyperbaric solutions of bupivacaine and morphine during cesarean section significantly reduced the incidence of postoperative shivering [7], while other studies have reported conflicting results [8]. The study by Hoshijima et al. showed that remifentanil was associated with a higher incidence of postoperative tremor than alfentanil or fentanyl, but this difference was not significant as compared with sufentanil. The widespread use of sufentanil in clinical practice makes it necessary to provide evidence on its adverse effects and anesthetic effects [9]. Therefore, in view of the controversy and necessity of the current evidence, this study conducted a systematic review and meta-analysis to provide theoretical basis for clinical decision making by exploring the role of sufentanil for spinal anesthesia in cesarean sections.

## 2. Methods

### 2.1. Literature Search

Literature searches were performed using Medical Subject Headings (Mesh) search terms in databases such as PubMed, Embase, ScienceDirect, and OVID. The search keywords were (“Sufentanil” [Mesh Terms] OR “Bupivacaine” OR “Ropivacaine”) AND (“spinal analgesia” [Mesh Terms] OR “intrathecal analgesia” OR “subarachnoid analgesia”) AND (“efficacy” OR “adverse effect” OR “motor block” OR “sensory block” OR “shivering” OR “pruritus”).

### 2.2. Literature Screening

Inclusion criteria were as follows. (1) The type of study design was a randomized controlled trial. (2) The study population is pregnant women aged 18–45 undergoing cesarean section under spinal anesthesia. (3) The interventional group received spinal anesthesia by sufentanil, whereas the control group received placebo. (4) The primary endpoint of the study included at least one of the following categories: time to sensory block, time to motor block, time to sensory recovery, and adverse outcomes including nausea, vomiting, postoperative shivering, hypotension, and pruritus.

Literature exclusion criteria were as follows. (1) Route of administration other than subarachnoid (spinal anesthesia). (2) The study did not specify the standard for the dosage of the drug. (3) Sample size of the interventional group or the control group less than 20. (4) Non-articles, such as reviews, conferences, reviews, or case reports.

### 2.3. Document Data Arrangement and Evaluation

Two investigators independently screened and extracted the following data from the included literature: type of study (open-label or double-blind trial), country or region of the study population, year and author of the study, sample size of the interventional and control groups, standardized drug dose, time of sensory block, time of motor block, time of recovery of sensory block, and occurrence of adverse reactions. All included studies were assessed for risk of bias by two independent investigators using the Cochrane risk of bias tool for systematic reviews and meta-analyses of randomized controlled trials [10] that included following aspects: (1) random number generation method (selection bias); (2) group concealment (selection bias); (3) blinding of investigators and subjects (implementation bias); (4) blinding (detection bias) to the primary endpoint measure; (5) integrity of research results and data; (6) selective reporting; and (7) other biases. The screening of studies was performed by first reading the title and abstract. Full texts of potentially eligible articles were then downloaded for further screening. When the two investigators were in dispute, a consensus was reached by discussing with a third researcher.

### 2.4. Statistical Methods

STATA17.0 (SE) was used for statistical analysis. The primary endpoint was the relative risk (RR) for categorical variables and the mean ± standard deviation for continuous variables. The random-effects model or fixed-effects model was used to combine risk estimates, as appropriate. The inter-study heterogeneity was assessed using Cochran's *Q* test. If there was significant heterogeneity between studies (I^2^>50%), random-effects model was used; otherwise, a fixed-effects model was used. Publication bias was described by funnel plots and assessed using Egger's and Begg's tests. All statistical results in this study were considered statistically significant at two-sided *P* < 0.05.

## 3. Results

### 3.1. Search Results and Literature Features

**Figure 1:**
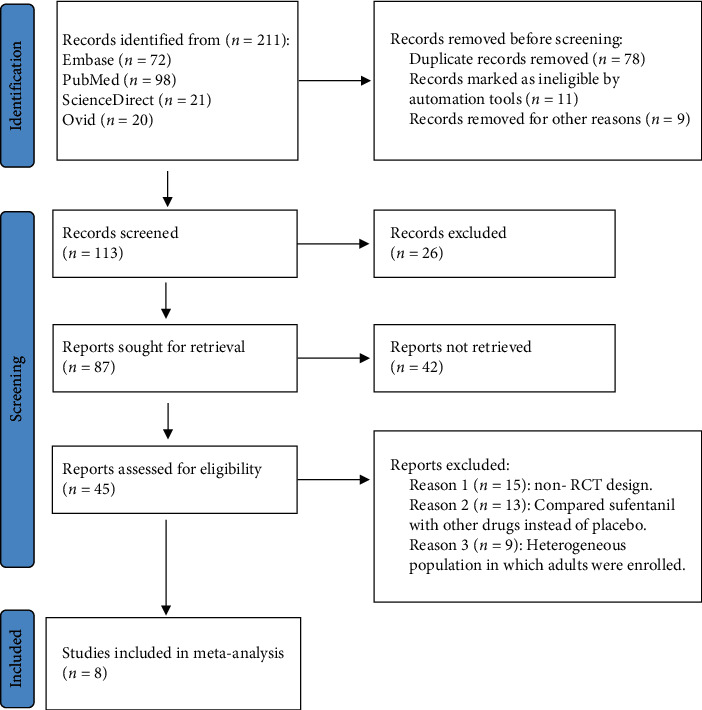
PRISMA flowchart: the process of screening literature for inclusion in meta-analysis.

A total of 211 related studies were generated. Finally, a total of 8 studies with a total of 503 women were included in the meta-analysis. The detailed process of literature retrieval and screening is shown in the PRISMA flowchart in Figure 1. The characteristics of the 8 included papers are shown in Table 1. The included studies were all randomized controlled trials, of which 4 reported the sensory block time, 2 reported motor block time, and 4 reported sensory recovery time. Adverse events of postoperative nausea and vomiting, hypotension, postoperative shivering, and postoperative skin pruritus were reported in 8, 4, 5, and 7 studies, respectively. Assessment of risk of bias by the Cochrane systematic review system found that 3 studies had possible selection bias of random number generation, 4 studies had possible selection bias of grouping concealment, and 2 studies had obvious grouping concealment selection bias; 4 studies had obvious selection bias of grouping concealment; 1 study had possible bias in blinding of researchers and subjects; 2 studies had possible bias in outcome measurement, 3 studies had obvious outcome measurement bias; 2 studies had possible bias in research results and data integrity; 4 studies had selective reporting bias; 2 studies had other possible biases.

### 3.2. Sensory Block Time

**Figure 2:**
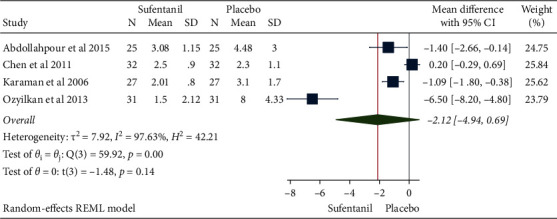
Forest plot of time to sensory blockade by sufentanil in spinal anesthesia.

**Figure 3:**
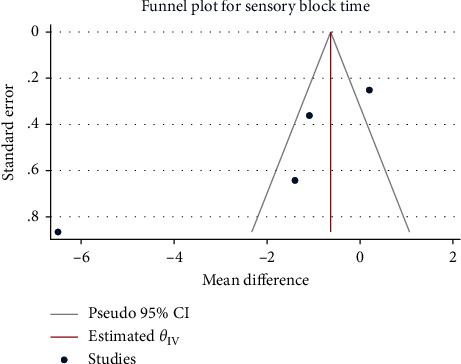
Funnel plot of time to sensory blockade by sufentanil in spinal anesthesia.

A total of 230 pregnant women from 4 studies were included. The results of the heterogeneity test were *H*^2^ = 42.21, I^2^ = 97.63%, and *P* = 0.00, indicating a high degree of heterogeneity. Mean differences were combined using random-effects models that showed no significant reduced onset time of sensory block for subarachnoid administration of sufentanil as compared with the control group (MD = -2.12, 95%CI: -4.94, 0.69, *P*=0.14), as shown in Figure 2. The funnel plot showed no obvious publication bias (Figure 3).

### 3.3. Sensory Recovery Time

**Figure 4:**
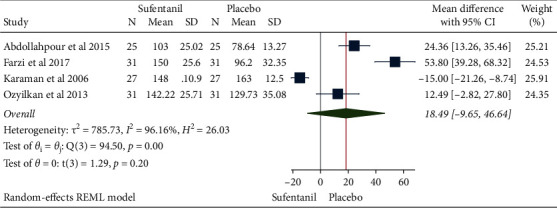
Forest plot of sufentanil on sensory recovery for spinal anesthesia during cesarean section.

**Figure 5:**
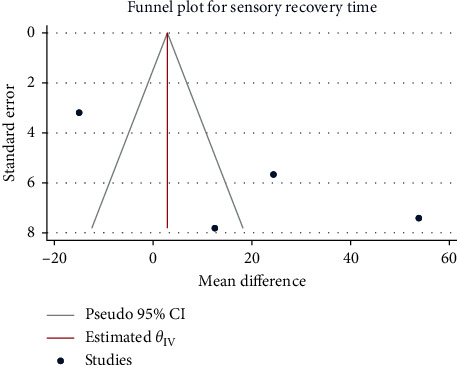
Funnel plot of sufentanil on sensory recovery for spinal anesthesia during cesarean section.

A total of 4 studies with 208 pregnant women were included. Mean differences were combined using random-effects models in the presence of high heterogeneity (*H*^2^ = 26.03, I^2^ = 96.16%, *P* < 0.001). The results showed that compared with the control group, subarachnoid administration of sufentanil for anesthesia did not significantly prolong the sensory recovery time (MD = 18.49, 95%CI: -9.65, 46.64, *P*=0.20, Figure 4). Potential publication bias was suggested by the funnel plot (Figure 5).

### 3.4. Occurrence of Postoperative Nausea

**Figure 6:**
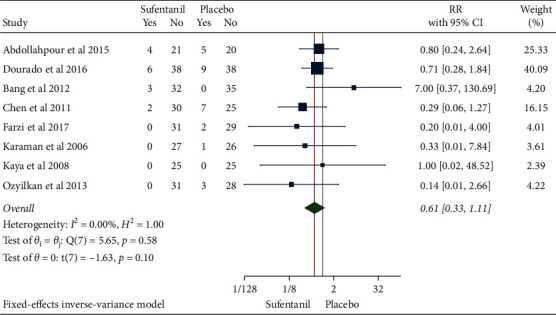
Forest plot of sufentanil on postoperative nausea after spinal anesthesia during cesarean section.

**Figure 7:**
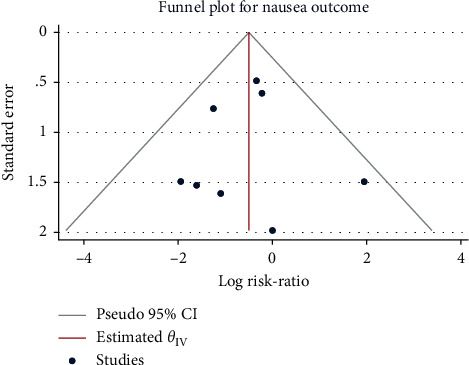
Funnel plot of sufentanil on postoperative nausea after spinal anesthesia during cesarean section.

A total of 503 patients in 8 studies were included. RR was then combined using a fixed-effects model based on the inverse variance method since the heterogeneity test indicated a low degree of heterogeneity (*H*^2^ = 1.00, I^2^ = 0.00%, *P*=0.58). Compared with the control group, the use of sufentanil did not significantly reduce the risk of postoperative nausea (RR = 0.61, 95%CI: 0.31, 1.11, *P*=0.10), as shown in Figure 6. The funnel plot showed no obvious publication bias (Figure 7).

### 3.5. Incidence of Postoperative Vomiting

**Figure 8:**
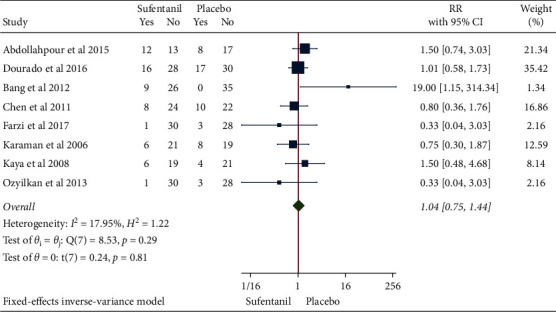
Forest plot of sufentanil on vomiting after spinal anesthesia during cesarean section.

**Figure 9:**
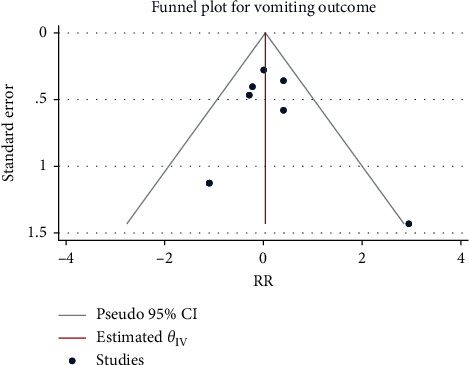
Funnel plot of sufentanil on vomiting after spinal anesthesia during cesarean section.

Pooled analysis in 503 patients from 8 studies using the fixed-effects model (*H*^2^ = 1.22, I^2^ = 17.95%, *P*=0.29) indicated that sufentanil did not significantly increase the risk of postoperative vomiting as compared to the control group (RR = 1.04, 95% CI: 0.75, 1.44, *P*=0.81, Figure 8). There was no obvious publication bias (Figure 9).

### 3.6. Postoperative Hypotension

**Figure 10:**
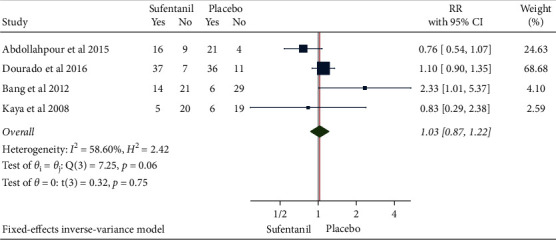
Forest plot of sufentanil on hypotension after spinal anesthesia during cesarean section.

**Figure 11:**
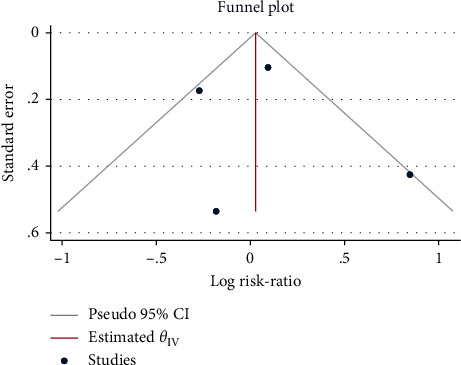
Funnel plot of sufentanil on hypotension after spinal anesthesia during cesarean section.

The results of the meta-analysis using the fixed-effects model (*H*^2^ = 2.42, I^2^ = 58.60%, *P*=0.06) in 261 patients from 4 studies showed that as compared with the control group, the use of sufentanil did not significantly increase the risk of postoperative hypotension (RR = 1.03, 95% CI: 0.87, 1.22, *P*=0.75, Figure 10). No obvious publication bias was detected (Figure 11).

### 3.7. Occurrence of Postoperative Shivering

**Figure 12:**
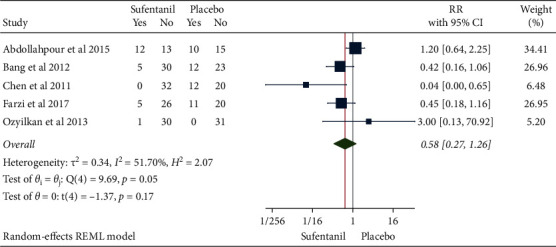
Forest plot of sufentanil on postoperative shivering after spinal anesthesia.

**Figure 13:**
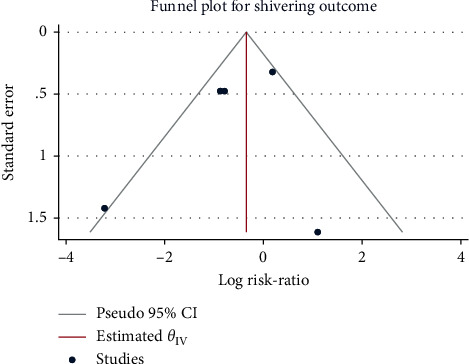
Funnel plot of sufentanil on postoperative shivering after spinal anesthesia.

A total of 5 studies with 308 patients were included. After confirming moderate heterogeneity (*H*^2^ = 2.07, I^2^ = 51.70%, *P*=0.05), the mean difference calculated using the random-effects model suggested that the use of sufentanil did not significantly increase the risk of postoperative shivering (RR = 0.58, 95% CI: 0.27, 1.26, *P*=0.17, Figure 12). The funnel plot (Figure 13) indicated no obvious publication bias.

### 3.8. Occurrence of Postoperative Skin Itching

**Figure 14:**
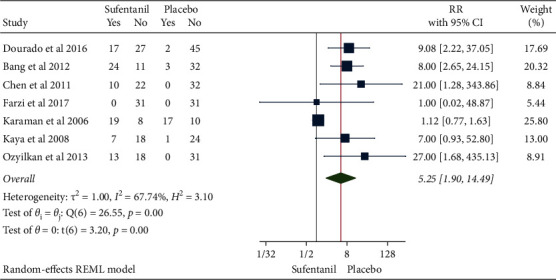
Forest plot of sufentanil on pruritus after spinal anesthesia during cesarean section.

**Figure 15:**
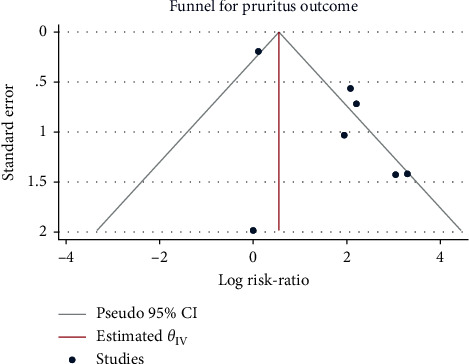
Funnel plot of sufentanil on pruritus after spinal anesthesia during cesarean section.

The results of the heterogeneity test from 8 studies with a total of 503 patients were *H*^2^ = 3.10 and I^2^ = 67.74% (*P* < 0.001), indicating moderate heterogeneity. Compared with the control group, the use of sufentanil significantly increased the risk of postoperative skin itching (RR = 5.25, 95%CI: 1.90, 14.49, *P* < 0.001, Figure 14). No obvious publication bias was observed (Figure 15).

## 4. Discussion

In order to achieve the ideal anesthetic effect during cesarean section, anesthesia that effectively covers the S2–S4 to T4–T12 segments of the spinal cord by blocking the sympathetic nerve is usually necessary. However, postpartum hypotensive adverse reactions were common [19]. The usual dosing regimen for local anesthesia for cesarean section is bupivacaine 0.5% (7.5–15 mg) [19], with some guidelines suggesting 10 mg bupivacaine alone or 8 mg bupivacaine in combination with other opioids (e.g., sufentanil) to be low doses [20], while other investigators consider bupivacaine to be low as long as its dose does not exceed 8 mg [21]. Due to the existing inter-study heterogeneity in terms of the definition of low dose, this study did not explore the source of heterogeneity by performing subgroup analysis according to dosages.

Previous studies have shown that many opioids, including sufentanil, are involved in inhibiting the release of cholinergic neurotransmitters [22, 23]. In addition, opioids can induce nausea, vomiting, hypotension, skin itching, and other adverse drug reactions by reducing the transmission of cholinergic neurotransmitters in the heart, gastrointestinal tract, and respiratory tract in the peripheral nervous system [24–26]. The research of Silva et al. showed that the target of respiratory depression caused by opioids is in the ventrolateral area of the rostral medulla oblongata [27]. In addition, many electrophysiological, anatomical, and pharmacological studies have shown that there are superficial and deep layers of the ventrolateral area of the rostral medulla oblongata, where many opioid-sensitive neurons and neurons associated with respiration and sympathetic output reside [28–30]. A 5 mg dose of sufentanil has been reported to be significantly associated with increased incidence of hypotension during cesarean delivery via the subarachnoid route [13].

Although meta-analysis [31] has demonstrated that bupivacaine combined with sufentanil reduced pain and shortened the duration of sensory block in women, it was associated with elevated incidence of pruritus. Nonetheless, the results of this study showed that subarachnoid administration of sufentanil numerically shortened the onset time of sensory block and motor block and prolonged the total onset time of local anesthesia; the difference was not statistically significant. On the contrary, the incidence of adverse drug reactions significantly increased, such as skin itching. Therefore, the superiority of this drug is still controversial and its clinical administration entails prudence. Since only 4 studies were included in the meta-analysis, the possibility of a potential bias associated with small sample size cannot be definitively ruled out. Consistent with our study results, other investigations have also reported that subarachnoid administration of 2.5ug–10ug sufentanil was not significantly associated with intraoperative hypotension [19, 21, 32].

This study has several limitations. First, although the quality of all included literature in this study was at least moderate, not all were free of risk of bias. Second, sample size calculation was reported in only a subset of publications, so it remains unclear whether the research results have sufficient statistical power to ensure reliability. In addition, the wide confidence interval of some endpoint indicators increased the uncertainty of statistical inference. At last, due to the geographic differences in clinical practice, the dosage of sufentanil differs significantly between studies, which may also introduce bias.

In conclusion, there was no significant difference in the onset time of sensory block, motor block, and the combined effect between subarachnoid and local administration of sufentanil. The incidence of some adverse reactions such as postoperative nausea, vomiting, hypotension, and tremor had no significant difference in the combined effect size. However, the incidence of skin pruritus was significantly increased, and the difference was statistically significant. Therefore, in the clinical use of this drug, it is still necessary to use it with caution in combination with the actual situation.

## Figures and Tables

**Table 1:** Characteristics of the 8 included papers.

Author	Study design	Sample size intervention /control	Age range	Time to sensory block	Time to motor block	Time to sensory recovery	Vomiting	Nausea	Hypotension	Shivering	Pruritus
Abdollahpour et al. [11]	RCT	25/25	27.33 ± 3.99	3.08 ± 1.15	4.48 ± 3	1.6 ± 0.7	3.84 ± 1.28	103 ± 25.02	78.64 ± 13.27	12/8	4/5
Dourado et al. [12]	RCT	44/47	26.02 ± 6.36	N/A	N/A	N/A	N/A	N/A	N/A	16/17	6/9
Bang et al. [13]	RCT	35/35	33.1 ± 2.5	N/A	N/A	N/A	N/A	N/A	N/A	9/0	3/0
Chen et al. [14]	RCT	32/32	28 ± 3	2.50 ± 0.9	2.31 ± 1.1	N/A	N/A	N/A	N/A	8/10	2/7
Farzi et al. [15]	RCT	31/31	26 ± 6.36	N/A	N/A	N/A	N/A	150 ± 25.6	96.2 ± 32.35	1/3	0/2
Karaman et al. [16]	RCT	27/27	30 ± 4.45	2.01 ± 0.8	3.1 ± 1.7	N/A	N/A	148 ± 10.9	163 ± 12.5	6/8	0/1
Kaya et al. [17]	RCT	25/25	28.6 ± 5.77	N/A	N/A	N/A	N/A	N/A	N/A	6/4	0/0
Ozyilkan et al. [18]	RCT	31/31	30.38 ± 5.6	1.52 ± 2.12	8 ± 4.33	3.75 ± 4.42	10 ± 5.1	142.22 ± 25.71	129.73 ± 35.08	1/3	0/3

## Data Availability

The data used to support the findings of this study are included within the article.
